# Errata

**Published:** 1816-06

**Authors:** 


### MONTHLY CATALOGUE OF MEDICAL BOOKS.

The Modern Practice of Physic, exhibiting the Characters, Causes, Symptoms, Prognostics, Morbid Appearances, and improved Method of Treating the Diseases of all Climates. By Robert Thomas, M.D. 8vo. Fifth Edition, revised and enlarged.

A Treatise on the External Chemical and Physical Characters of Minerals. By Robert Jameson, Professor of Natural History, &c. &c. &c. 8vo. Second Edition.

Pharmacopœiarum Collegiarum Regalium, Londini, Edinburgi, et Eblanæ, Conspectus Medicus; à Edwardo Goodman Clarke, M.D. Col. Reg. Med. Lon. Exercitusq. Medico. etc. 18mo. Edit. alteria.

A System of Physiological Botany. By the Rev. P. Kieth, F.L.S. Illustrated by nine Engravings; in 2 vols. 8vo.

Observations and Inquiries into the Nature and Treatment of the Yellow, or Bulam Fever, in Jamaica and at Cadiz; particularly in what regards its Primary Cause and assigned Contagious Powers: illustrated by Cases and Dissections, with a View to demonstrate that it appears divested of those Qualities assigned to it by Mr. Pym, Sir J. Fellowes, and others. In a Series of Memoirs. By Edward Doughty, Member of the Royal College of Surgeons of London, and Surgeon to the Forces. 1 vol. 8vo.

---

### *NOTICES TO CORRESPONDENTS.*

*We cannot admit the Letter from our ingenuous Norfolk Correspondent, unless he will permit us to shorten it in some parts.*

*We cannot admit an Anonymous Paper on the practice of a Gentleman who always gives us his name. Still less can we undertake to circulate reports, which are always current enough without our adding to their publicity. The story of the Master of an Inn may do very well for a sapient shrug among the inhabitants of a country town, but to that it should be confined.*

*Communications have been received from* John Hoare, Esq., Medicus, One of the Fraternity, W. S., *&c. &c.*

*Our Correspondents will please to be particular in the Address of their Communications, which should be thus,* "To the Editors of the London Medical and Physical Journal, at Mr. Souter's, No. 1, Paternoster-Row; or at Mr. Adlard's, 23, Bartholomew-Close."

---

ERRATA.

Page 400, line 11 from the bottom, for *author* read *patient.*
———— 9 ————, for *testaceæ* read *testacea.*
—— 404, —— 6 ————, for *thee vacuations* read *the evacuations.*
—— 413, —— 16 ————, for *ingenious* read *ingenuous*
—— 424, —— 12 ————, for *influence* read *atmosphere.*

### INDEX

### TO THE THIRTY-FIFTH VOLUME.

ABDOMEN, case of wound of the 222
———, fœtus found in that of a girl 411
Abscess, case of 346
Accoucheurs, remarks on 84
Acids in syphilis, effects of 72
Acidum sulphuricum in typhus, efficacy of 73
Act, remarks on the Apothecaries' 19
———, extract from the Apothecaries' 20
Acute rheumatism, Mr. Ronalds on 8
Adams, Dr. on midwives and accoucheurs 84
———, on Dr. Calvert's account of the plague at Malta 323
Almanack, Bill of Mortality in the St. Petersburgh 163
Alum beneficial in tympanites 73
ANALYSIS CRITICAL, of all new medical publications 41, 119, 217, 315, 398, 478
Anatomy of the human body, Mr. Armiger on the 338
Aneurism, case of carotid 147
———, Dr. Whitridge's case of inguinal and popliteal 368
———, on the cure of 411
———, case of axillary 412
Animal heat, Dr. Hale on, in answer to Mr. Brodie 291
Apothecaries' Act, remarks on the 19
——— Repository, Mr. Rootsey on the review of his book in the 275
——— Bill, memorial of the 334
———, report of their committee 427
———, resolutions of the 488
Armiger, Mr. on the anatomy and physiology of the human body 508
Arsenic, the effects of 338
Arterial pulse, Dr. Parry on the 478
Arteries, on the application of ligatures to 190
Ascites, Mr. Gay's case of 434
Assalini, Chev. his instruments approved 396
Atkinson, Mr. on the obstetric practice 3
———, Dr. Kinglake's reply to 174
Atmosphere, its state considered the cause of hospital gangrene 20
Baillie, Dr. on the dedication of Dr. Roberton's book 465
Barbadoes leg, case of 392
Bark, improved mode of exhibiting, in agues 253
Barlow, Mr. on his improved sound 81
Bartlett, Dr. his remarkable extra-uterine case 278
Bath, description of a sun 159
Bayle, Death of Dr. 513
Bethlem, on the election of physicians and apothecary at 511
Bichat's physiological researches on life and death, review of 41, 125
Bill of mortality for 1815, remarks on 162
——— in Russia 163
Boney substance extracted from the rectum of a young man 108
Bladder, Mr. Wadd on diseased 217
Bleeding in peripneumony, Dr. Kinglake on 105
——— beneficial in hydrophobia 515
Blood, Dr. Carson on its motion 5[illegible]
Bloodletting, questions concerning 381
——— in fever, Dr. Dickson on 499
Blow-pipe, Mr. Brooke's improved 510
Botanical lectures, Mr. Salisbury's account of his 430, 516
BOOKS, announcement of new 75, 166, 342, 515
———, catalogue of medical 80, 168, 256, 344, 432, 520
Brain, on the death of the 138
———, Dr. Powell on the pathology of the 295
Brodie, Mr. reply to 291
Bronchotomy, success of, in croup 417
Brugman's, Prof. on the cause of hospital gangrene 20
Bulam fever, Dr. Pym on the 507
Calton, Mr. his case of wound of the abdomen 222
Calvert, Dr. on the plague at Malta 319
Cancer uteri, Prof. Osiander's remedy for 426
Caribbees, on the diseases of the inhabitants of the 475
Carpue, Mr. on his revival of the Taliacotian art 293

Carson, Dr. on the motion of the blood 55
Cases—of a large incysted tumour, connected with the kidneys 1
——— acute rheumatism 8
——— violent cephalalgia 9
——— puerperal fever, with dropsy 14
——— chronic hydrocephalus 72
——— demonomania 74
——— death from swallowing a cocoa nut 100
——— bony substance extracted from the rectum of a young man 108
——— carotid aneurism 147
——— malformation of the heart ib.
——— the effects of tobacco 151
——— obstinate struma 161
——— pulmonary disease 183
——— polydipsia 199
——— true elephantiasis 210
——— fracture of the skull 222
——— wound of the abdomen ib.
——— puerperal fever ib.
——— inflammation of the heart 223
——— inguinal and popliteal aneurism 235
——— head-ach, occasioned by worms 252
——— extra-uterine fœtus 278
——— illustrative of the pathology of the brain 295
——— abscess 347
——— erysipelas ib.
——— inguinal aneurism 368
——— Barbadoes leg 392
——— locked jaw 408
——— incontinence of urine 411
——— aneurism ib.
——— axillary aneurism 412
——— cynanche laryngea 413
——— croup 417
——— ascites 434
——— enteritis 436
——— rupture of the uterus 449
——— placental presentation 462
——— small-pox after vaccination 477
——— insanity 495
——— somnolency 509
——— syphilis 512
——— hydrophobia 515
Caterpillars, singular phenomenon in some 337
Cauterizing, ancient method of 387
Cephalalgia, violent case of 9
Chalk-cliff, account of a living hog found in a 511
Channing, Dr. on spontaneous hæmorrhage 450
Chemistry, on Mr. Crowe's elementary View of 59
Chemistry, on the discoveries in 111
Child affected by mercury whilst suckling 512
Children, Dr. Clarke on the diseases of 235
Chocolate-nut, death occasioned by swallowing one 100
Cholic, on the free use of opium in 349
Chopart's operation, account of 74
Christ's Hospital, diseases of the children at 424
Chronic hydrocephalus, case of 72
City of London Truss Society, report of the 246
Clanny Dr. account of his lamp for coal mines 243
Clarke, Dr. on the diseases of children 235
Cline, Mr. account of his Hunterian oration 249
Collectanea Medica, 20, 109, 204, 295, 381, 466
Congenital hernia, discoveries concerning 109
Contagion, observations on 267
Correspondents, notices to 80, 168, 256, 344, 432, 520
Craniology, Mr. Forster's remarks on 72
Crawford, Mr. his case of fracture of the skull 222
Critical Analysis 41, 119, 217, 315, 398, 478
Croup, on bronchotomy in 417
Crowe, Mr. his elementary view of chemistry 59
Cupping and leeching, questions concerning 383
Cynanche laryngea, case of 413
Dalrymple, Mr. his case of aneurism 411
Davy's, Sir H. on a solid compound of iodine and oxygen 113
Deafness, on piercing the tympanum in 253
Death of the brain, its influence on the body 138
——— of Dr. Bayle 513
——— by swallowing a cocoa-nut 100
——— distinguished by Mr. Hunter from cessation of action 126
Demonomania, case of 74
Denman, Dr. memoirs of 62
Diarrhœa, remedy in cases of 73
Dickson, Dr. on bloodletting and purgatives in fever 499
Diseases, report of 79, 168, 254, 342, 432, 518
——— of children, Dr. Clarke on the 235
——— in Christ's Hospital 424
——— on pulmonary 182
——— of the inhabitants of the Caribees 475
Dissections 148, 223, 230, 231, 232, 339, 410, 438
Drugs, London prices of 77
Duncan, Dr. his cases of inflammation of the heart 223

Dunn, Mr. on puerperal fever 222
Dunninald, account of the sleeping woman at 509
Earle, Mr. on nicotiana in retention of urine 408
Edgell, Dr. on the obstetric practice 350
———— on a placental presentation 462
Edinburgh Medical and Surgical Journal, review of the 147, 222, 398, 499
Editors, their remarks on vaccination 98
————, hints to the 266
————, reply to the 267
————, defence of the review of Dr. Philip's paper 315
Elephantiasis, two cases of true 210
———— of the moderns, case of 392
Emetic tartar, Mr. Hume on 514
————, Dr. G. Pearson on 346
Enteritis, Dr. Francis's case of 436
Entomology, extracts from Kirby and Spence's 466
Epilepsy, cure for 337
Erysipelas, case of 347
Euclid, translation of all the works of 245
Eyes, chronic inflammation of, cured by vaccination 161
Fever, Dr. Thacher on puerperal, with dropsy 14
————, Dr. Dunn on puerperal 222
————, bark efficacious in intermittent 253
————, on the typhoid state of 353
————, ancient treatment of 475
————, on bloodletting and purgatives in 499, 505
————, at Gibraltar, Mr. Humphreys on the 505
————, Dr. Pym on the Bulam 507
Field, Mr. on the diseases of children at Christ's Hospital 424
Fiske, Dr. his cases of abscess and erysipelas 347
Flora of St. Helena, on a new 337
————, Mr. Salisbury's calendar of 430, 516
Fœtus found in the abdomen of a girl 411
Foster, Mr. on the heads of insane persons 72
Fracture of the skull, case of 222
France, Royal Institute of 245, 423
Francis, Dr. on a case of enteritis 434
Gall, Dr. remarks on his lectures 251
Gangrene, on the cause of hospital 20
Gaye, Mr. on a remarkable case of ascites 434
Gecko, on the feet of the 333
Gestation, healthy child born in the fifth month of 424
Girl, fœtus found in the abdomen of a 411
Gibraltar, on the fever at 505
Gout, Mr. Norman on cold applications in 203
Guadaloupe, account of its ancient inhabitants 214
Guinea-worm, on the generation of the 335
Hale, Dr. on animal heat 291
Hæmorrhage, Dr. Channing on spontaneous 450
Heart, case of malformation of the 147
————, on inflammation of the 223
————, remarks on its actions 260
Heath, Mr. on the generation of the guinea-worm 335
Helena, St. new flora of 337
Henry, Mr. on a boney substance extracted from the rectum of a young man 108
Hernia congenita, discoveries in 109
Hill, Mr. on the cure of insanity 488
———— cause of insanity 495
Hog, account of the hybernating 511
Home, Sir Everard, and Spurzheim, their opinions confirmed 251
Hosack, Dr. on the typhoid state of fever 353
Hospital gangrene, Prof. Brugman on the state of the atmosphere as the cause of 20
————, report of the small-pox 99
————, meetings of ditto 203
———— at Vienna, appearance of spinodorsitis in the 339
Hottentot Venus, death of the 259
Human body, Mr. Armiger on the anatomy and physiology of the 508
Hume, Mr. on the emetic tartar 514
Humphreys, Mr. on the fever at Gibraltar 505
Hunter, Mr. anniversary of, celebrated 249
————, his doctrines too little known on the continent 72
Hydrencephalus, Dr. Yeats's remarks on 88
Hydrophobia, bleeding beneficial in 515
Hyslop, Mr. on incontinence of urine 411
Ileum, malformation of the 438
Incontinence of urine, case of 411
Inflammation of the heart, on 223
———— of the eyes, benefit of vaccination in 161
Insane persons, Mr. Foster on the heads of 72
Insanity, case of 495
————, cure for 337-8
————, Mr. Hill on 448
Insects, on those inhabiting the human body 466
Insolation, remarks on 160
Institute of France, proceedings of the 245, 423
Institution for administering medical aid to sick poor 328
Instruments, testimonials of Chev. Assalini's 396

Instruments for ligatures, account of a new 199
—— for extracting teeth 433
INTELLIGENCE, Medical and Philosophical 66, 155, 243, 331, 422, 509
—— foreign 73, 156, 252, 337
Inoculation, case of small-pox several years after 477
Iodine and oxygen, Sir H. Davy on a solid compound of 113
Jackson, Mr. on a large incysted tumour connected with the kidneys 1
Jones, Dr. on his discovery in ligatures 193
Kidneys, case of a large incysted tumour connected with the 1
Kinglake, Dr. reply to 3
—— on bleeding in peripneumony 105
—— on the Obstetric practice 174
—— replies to 282, 348
—— Mr. Wayte on the opinions of 359
—— his reply to Dr. Merriman 363
Kirby and Spence's Entomology, extracts from 466
Lamp for mines, Dr. Clanny's account of his 243
——, account of Sir H. Davy's and Mr. Stephenson's 336, 516
Langres, shower of stones near 423
Latham, Dr. letter to, on emetic tartar 514
Lanterns, on a method of making ship 336
Lawrence, Mr. on a case of elephantiasis of Aretæus 210
Leeches, Dr. O'Berne on applying 275
——, on cutting off the tails of 352
LECTURES announced.—Dr. Spurzheim on Anatomy, 72.—Dr. Adams on the institutes and practice of Medicine, 76, 427.—Mr. Taunton on Anatomy, 76.—At St. Bartholomew's Hospital, ib.—Mr Singer on electricity, &c. ib.—Dr. Clutterbuck on the theory and practice of Physic, ib.—Dr. Clarke on Midwifery, ib.—Dr. Squire on Midwifery, 76, 341.—Mr. Bell on Surgery, 76.—At St. Thomas' and Guy's Hospitals, 165.—At the Russel Institution, 166.—Dr. Merriman on Midwifery, ib.—Mr. Fox on the Teeth, 254.—Mr. Clarke on Midwifery, ib.—Mr. Lawrence on Anatomy, 341.—Dr. Clutterbuck on the practice of Physic, &c. ib.—Mr. Salisbury on Botany, 430, 516.—Dr. Carpue on Anatomy, 515.—Dr. Squire and Dr. Davis on Midwifery, ib.
Lepers, ancient mode of examining 313
Leprosy, tokens of 311
Lettsom, Dr. J. C. memoirs of 204
Lice, on a species of 466
Life and Death, X. Bichat on 41, 125
Ligatures, on the application of, to arteries 190
——, Ambrose Parê's claim to the invention of 192
——, Dr. Jones's discovery in 193
——, description of a new one 199
Linnæan Society, proceedings of the 244, 511
London price of Drugs 77
—— Pharmacopœia, Dr. Pearson on the 77
—— anniversary of the Medical Society of 333
Longevity in St. Petersburgh, instances of 163
Mad-houses, Report of the Committee on 28
Madness, Mr. Hill on 488
Malformation of the heart, case of 147
—— ileum 438
Malta, Mr. Stafford on the plague at 152
——, Dr. Calvert on ditto 319
Mania, Mr. R. Walker on 269
Mantell, Mr. on an hybernating hog 511
Medicines, on the action of specific 422
Medical and Philosophical INTELLIGENCE 66, 155, 243, 331, 422, 509
—— Benevolent Society, resolutions of the 161
——, account of the 246
——, permanency of it 509
—— aid to sick poor, institution for admistering, review of the 328
—— topography of New-Orleans, on the 398
—— Society of London, anniversary of 333
Medico-Chirurgical Transactions, review of 319, 408
Memoirs of Dr. T. Denman 62
—— of Dr. J. C. Lettsom 204
Mercury, on the use and abuse of 270
——, a child affected by, whilst suckling 512
Merriman, Dr. on the practice of Midwifery 282
—— reply to 363
Metastasis, singular instance of 269
Meteorological Register 78, 167, 255, 343, 431, 519
Midwifery, Dr. Merriman on the practice of 282
Midwives and accoucheurs, Dr. Adams on 84
Milk, proposed as a remedy for the poison of certain mushrooms 72
Millington, Mr. on small-pox succeeding vaccination 477
Mortality, remarks on the general bill of 162

Mushrooms, milk proposed as a remedy for the poison of certain 72
Nail, on the re-production of the 426
Nasal operation, on the revival of the 294
Navy, Dr. Trotter on the health of the 259
Nervous and electric fluid, Mr. Purton on the 104
—— sanguiferous systems, Dr. Philip on 119, 331
New-Orleans, medical topography of 398
Nicotiana, use of it in retention of urine 408
Nitrate of silver, on the 337
Norman, Mr. on the cold infusion in gout 203
Notices to Correspondents 80, 168, 256, 344, 432, 520
O'Berne, Dr. on the mode of applying leeches 275
Obstetric practice, Mr. Atkinson on 3
——, Dr. Adams on 84
——, Mr. Starr on 348
——, Mr. Edgell on 350
——, Mr. Wayte on 359
——, Dr. Kinglake on 363
Oil-skin and oiled silk, on its application to the human body 169
Opium in colic, free use of 349
Osiander, Prof. his remedy for cancer uteri 426
Oxford, Mr. R. Walker on vaccination in 93
Palsy, a cure for the 338
Paré, on his claim to the invention of ligatures 192
Parry, Dr. on the arterial pulse 478
Pearson, Dr. G. on the London Pharmacopœia 345
Peripneumony, Dr. Kinglake on bleeding in 105
Petersburgh Almanack, abstract of the 163
Pharmacopœia, Dr. G. Pearson on the London 345
Philosophical Transactions of the Royal Society, review of 119
Philip, Dr. on the nervous and sanguiferous systems 119, 331
—— editor's defence of the review of his paper 315
—— his vindication of the identity of galvanism and the nervous influence 423
—— on the review of his paper 460
Phillips, Dr. his case of locked-jaw 408
—— on a case in which parts of a fœtus were found in the abdomen of a girl 411
Physiological researches on life and death, M. Bichat's 41, 125
Placental presentation, case of 462
Plague at Malta, Mr. Stafford on the 152
——, Dr. Calvert on the 319
Plantarum, a new species of 511
Polydipsia, Mr. Ware's case of 199
—— singular cases of 201
Powell, Dr. the evidence of, to the committee on mad-houses 28
—— on the pathology of the brain 295
Prescott, Mr. his case of death from swallowing a chocolate-nut 100
Prices of drugs 77
Prize Questions announced, at Paris 70
——, at Rouen ib.
——, at Haerlem ib.
——, at Copenhagen 71, 341
——, at Norway 341
——, Romford, voted to Dr. Wells 337
Prosecution against Mr. Taunton, remarks on the 66, 155
—— Dr. Walker on the 203
Puerperal fever, with dropsy, case of 14
Pulmonary diseases, Dr. Sutton on 182
Pulse, Dr. Parry on the arterial 478
Pulvis antimonialis, Dr. Pearson on the 345
Purton, Mr. on the identity of the nervous and electric fluid 104
Pym, Dr. on the Bulam fever 507
Questionary of Cyrurgyens, extracts from the 311, 381
Ramsey, Dr. on oil-skin and oiled silk applied to the human body 169
Report of Diseases 79, 168, 254, 342, 432, 518
—— of the small-pox hospital 99
Review of Dr. Philip's paper, defence of 317
Re-production of the nail 426
Reviewer, Dr. Philip's remarks to the 461
Rheumatism, Mr. Ronalds on acute 8
——, success of the American Indian warm-bath in a case of 251
Roberton, Dr. remarks on the dedication of his book 465
Ronalds, Mr. on acute rheumatism 8
Rootsey, Mr. on the review of his work in the Apothecaries' Repository 275
——, his general dispensatory reviewed 141
Royal Institute of France, proceedings of the 245, 334
—— Medical Society of Copenhagen, transactions of 69
—— Society of Edinburgh, proceedings of the 509
—— of London, meetings of the 69, 243, 331, 422
——, transactions of the 119
——, election of the Archdukes John and Lewis as members of the 333
—— of Sciences at Copenhagen, proceedings of the 69
Rupture of the uterus, favourable termination of a case of 449
Safe-lamp, Dr. Clanny's account of his 243
——, on Sir H. Davy's and Mr. Stephenson's 336

Salisbury, Mr. his botanical lectures and excursions 430, 516
Sand-baths, effects of 338
Saraccenia flavia and adunca, account of the 244
Scarenger, death of, by apoplexy 70
Scurvy, Dr. Trotter on sea 257
———, remedy in 338
Secretion, on the nervous influence in 331
Ship-lanterns, on making 336
Silver, on the nitrate of 337
S. J. E. letter to, on some anomalous symptoms in the organs of speech 9
Skull, case of fracture of the 222
Sleeping woman of Dunninald, account of 509
Small-pox, on the progress of the natural 439
———, on the treatment of 445
———, occurrence of, several years after inoculation 477
——— Hospital, Mr. Wachsel's report from the 99
———, meeting of 203
Somnolency, case of protracted 209
Sound, Mr. Barlow on his improved 81
Southey, Dr. on true elephantiasis 213
Spasmodic complaints, cure for 161
Specific medicines, on the action of 422
Spinodorsitis, appearance of, in the Vienna hospital 339
Spurzheim, remarks on his treatment in England 251
Stafford, Mr. on the plague at Malta 152
Starr, Mr. on the obstetric practice 348
Stones, fall of, near Langres 423
Struma cured by burnt sponge 161
Sun-bath, description of a 159
——— beams, advantages of the, in certain diseases 157
Sutton, Dr. on pulmonary diseases 182
Syphilis, use of acids in 72
———, remedy in 74
———, case of 512
Taliacotian art, on the revival of, by Mr. Carpue 293
Taunton, Mr. on the prosecution against 66, 155
Teeth, account of Mr. Whitford's new instrument for extracting 433
Thacher, Dr. on puerperal fever with dropsy 14
Thompson, Mr. letter to, on a review of the Apothecaries' Repository 275
Tobacco, on the effects of 151
Todd, Mr. on the Torpedo 333
Tongue as a double organ, on the 250
Topography of New-Orleans, on the medical 398
Torpedo, Mr. Todd's remarks on the 333
Transactions of Medico-chirurgical Society reviewed 319, 408
——— of the Royal Society reviewed 119
Treatment of some extraordinary symptoms 9
Trotter, Dr. on sea-scurvy 257
——— on the health of the navy 259
Tumour, case of a large incysted 1
Tympanites, beneficial effects of alum in the fatal 73
Typhus, on the efficacy of acidum sulphuricum in ib.
——— castrensis, case of blue-nose attending 156
Vaccination, Mr. R. Walker on the state of, in Oxford 93
———, editors' remarks on 98
———, cure of chronic inflammation of the eyes, by 161
———, on small-pox appearing several years after 477
Venus, death of the Hottentot 252
Vienna, appearance of spinodorsitis in the hospital at 339
Ulcers, Mr. Whately on the cure of 418
Upas artiar, on the poison of the 330
Urine, use of nicotiana in incontinence of 408
———, case of incontinence of 411
Urtication, on the revived practice of 73
Uterus, rupture of the, favourably terminated 469
Uwins, Dr. appointment of 75
Wachsel, Mr. his report from the Small-pox Hospital 99
Wadd, Mr. on hernia congenita 109
——— his cases of diseased bladder and testicle 217
Walker, Mr. R. on vaccination 93
——— on mania 268
——— on the progress of the natural small-pox 439
——— on the treatment of do. 445
Ware, Mr. on a case of polydipsia 199
Warm-bath successful in rheumatism 251
Wayte, Mr. reply to 174
Wells, Dr. the Romford prize voted to 337
Whately, Mr. on the cure of wounds and ulcers on the legs 418
Whitford, Mr. his new tooth instrument 433
Whitridge, Dr. on inguinal aneurism 368
Woman, account of a sleeping 509

### PLATES.

Mr. Barlow's improved Sound for detecting the stone 32
Mr. Ramsey's new Vapour-bath - - 173
Instruments formerly used in cauterizing - . - 360
Mr. Whitford's new Instrument for extracting Teeth 433

