# Errata

**Published:** 1816-07

**Authors:** 


					P. 520, for
aheriu read altera.

				

